# Regional heterogeneity of malaria prevalence and associated risk factors among children under five in Togo: evidence from a national malaria indicators survey

**DOI:** 10.1186/s12936-022-04195-6

**Published:** 2022-06-03

**Authors:** Gountante Kombate, Wakpaouyare Gmakouba, Susana Scott, Komi Ameko Azianu, Didier Koumavi Ekouevi, Marianne A. B. van der Sande

**Affiliations:** 1 Society for Study and Research in Public Health, Ouagadougou, Burkina Faso; 2 Department of Public Health, Institute of Tropical Medicine, Antwerp, Belgium; 3 Ministry of Health, Public Hygiene and Universal Access to Care, Lomé, Togo; 4 Department of Infectious Diseases Epidemiology, London School of Hygiene & Tropical Medicine, London, UK; 5 Department of Public Health, University of Lomé, Lomé, Togo; 6 Julius Centre, Global Health, University Medical Centre Utrecht, Utrecht, The Netherlands

**Keywords:** Regional heterogeneity, Malaria prevalence, Children under 5 years, Togo

## Abstract

**Background:**

Malaria remains a major cause of morbidity and death among children less than 5 years of age. In Togo, despite intensification of malaria control interventions, malaria remained highly prevalent, with significant heterogeneity from one region to another. The aim of this study is to explore further such regional differences in malaria prevalence and to determine associated risk factors.

**Methods:**

Data from a 2017 cross-sectional nationally representative malaria indicator survey was used. Children aged 6–59 months in selected households were tested for malaria using a rapid diagnostic test (RDT), confirmed by microscopy. Univariate and multivariate logistic regression analysis were performed using Generalized Linear Models.

**Results:**

A total of 2131 children aged 6–59 months (1983 in rural areas, 989 in urban areas) were enrolled. Overall 28% of children tested positive for malaria, ranging from 7.0% in the Lomé Commune region to 4% 7.1 in the Plateaux region. In multivariate analysis, statistically significant differences between regions persisted. Independent risk factors identified were higher children aged (aOR = 1.46, 95% CI [1.13–1.88]) for those above 24 months compared to those below; households wealth quintile (aOR = 0.22, 95% CI [0.11–0.41]) for those richest compared to those poorest quintiles; residence in rural areas (aOR = 2.02, 95% CI [1.32–3.13]).

**Conclusion:**

Interventions that target use of combined prevention measures should prioritise on older children living in poorest households in rural areas, particularly in the regions of high malaria prevalence.

## Background

Malaria continues to be major public health problem. In 2020, a total of 229 million cases of malaria and 409,000 deaths were reported, with the vast majority occurring in sub-Saharan Africa (94% of malaria cases and 99% of deaths) [1]. The children under five years of age continue to be at greatest risk of severe malaria, accounting for 67% of all malaria deaths. Prevalence is influenced by environmental, vector and human-related factors [2].

In Togo, malaria is endemic with a high prevalence. In 2018, 2,002,877 cases and 905 deaths were recorded on a general population about 7 million inhabitants [3]. Children under five years of age represented 31.6% of cases. This overall prevalence hides a strong heterogeneity between the regions ranging from 2.6% in the common Lomé Commune region to 43.0% in the Savannah region [3]. The socio-economic burden on the population is significant. In one study, the average expenditure per household on malaria prevention measures was US$ 8, which represents 5–15% of monthly household income[4]. Through the Global Technical Strategy for Malaria Control (2016–2030), Togo is one of the 35 countries committed to eliminate malaria by 2030 [5]. Therefore, the Togolese government through the National Malaria Control Programme (Programme National de Lutte contre le Paludisme, PNLP) has developed five-year plans, the first of which (National Health Development Plan 2017–2022) builds on known high-impact interventions to control malaria in the country with the goal to move towards malaria elimination by 2030 [6]. The main strategies adopted in this plan are the reinforcement of mass distribution of long-lasting insecticide-treated nets (LLINs) introduced since 2008, chemoprevention of seasonal malaria (SMC) for children under 3 to 59 years of age introduced since 2013, and intermittent preventive treatment for pregnant women introduced since 2005 [6].

Despite these initiatives, malaria remains a challenge. A retrospective longitudinal study using PNLP routine data from 2008 to 2017, showed an average annual increase in malaria cases in children under five of + 13.1%, also with strong heterogeneity ranging from + 6.3% in the Lomé Commune region to + 16.7% in the Centrale region [7]. Several other studies on malaria in Togo have focused on the clinical management of cases [8, 9], the evolution of malaria incidence [7], the evaluation of the implementation of malaria control interventions [10, 11], clinical trials on malaria vector resistance to insecticides [29, 30] and socio-cultural factors [12]. So far, there are very few studies examining the risk factors associated with regional heterogeneity in malaria prevalence among children under five in Togo using national population-based survey data [7]. The aim of this study was to explore further such regional variation in malaria prevalence and to determine the associated risk factors.

## Methods

### Study context

**Fig. 1:**
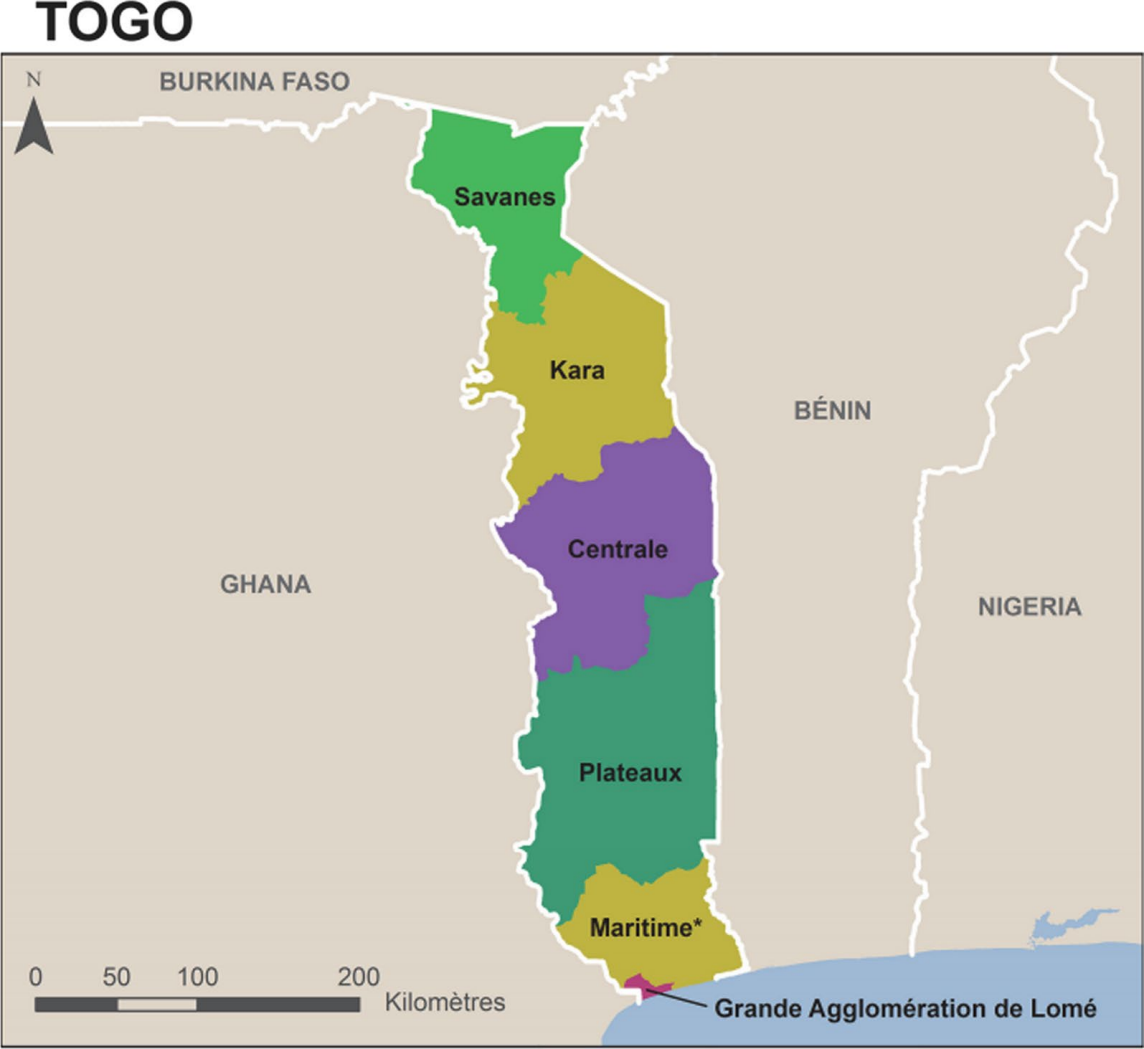
Togo administrative map (Ministry of Health 2017)

**Fig. 2:**
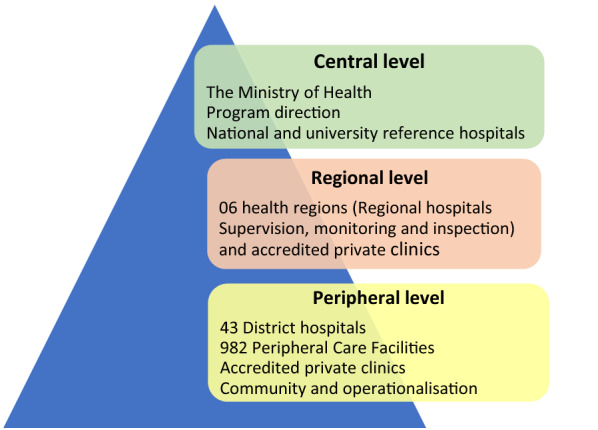
Togo's health system pyramid (Ministry of health, 2017)

Togo is a country in West Africa, with an population of about 7 million inhabitants in 2021, with a density of 152 inhabitants/km2 [13]. It borders Burkina Faso to the north, the Gulf of Guinea to the south, Benin to the east and Ghana to the west. The country is divided into six health regions and forty-three health districts in total (Fig. 1). The health system in Togo is organized as a three-level pyramidal structure [3] (Fig. 2). The first level is composed of the central administration and the different central departments and programmes where the guidelines and national policies are developed. The regional (or intermediate) level includes six health regions which provide coordination and technical support to the peripheral health districts. The third level is represented by the health district which is the most decentralized operational entity. There are 43 health districts and 944 peripheral health units.

### Study type and sample design

This was a secondary data analysis project using cross-sectional data from the Togo Malaria Indicator Survey (TMIS) 2017. These data provide information on malaria prevalence among children under five and pregnant women in the country [14]. The TMIS carried out a two-stage sampling method to select the sample. Using information from the last general population census in Togo in 2010 [15], each region was subdivided into Enumeration Areas (EAs). At the first step, 171 EAs were drawn with a probability proportional to the size and, 30 households were drawn randomly in each EAs selected. All women aged 15–49 reporting to usually live in the selected households or present the night before the interview with or without children under five were eligible to be interviewed. Selected households with neither a woman aged 15–49 nor a child under five were only included for the household questionnaires. In total, the sample consisted of 171 EAs, 5,130 households (1800 in urban areas and 3330 in rural areas), 4895 women from 15 to 49 years old (1684 in urban areas and 3,211 in rural areas), and 3271 children under five (2441 in rural areas and 830 in urban areas) [16].

### Tools and data collection

Three questionnaires were used in the context of the 2017 TMIS: a household questionnaire, a women questionnaire, and a biomarker questionnaire. The household questionnaire recorded all household members and visitors who slept there the night before the interviewer visited the household, water source, types of toilets, habitat characteristics, possession of durable goods, and use of mosquito nets were collected. The women questionnaire was used to collect information on socio-demographic characteristics, knowledge of malaria and prevention measures, births over the last 5 years, prevalence and treatment of fever in children under five. Finger prick blood samples for malaria testing were taken from all children aged 6–59 months in the selected households, for whom the parents or responsible adults had previously given their informed consent. Screening for malaria was done with a rapid diagnostic test (RDT), namely the SD Bioline Malaria Ag Pf/Pan with a sensitivity of 94.0% and specificity of 91.4%. Children who tested positive for malaria, or who had other signs of severe malaria or other serious illnesses, were referred to the nearest health facility for "advice and action" following the national health policy in Togo. Blood collection on slides was carried out to confirm the infection status of all children using a microscope. After drying and fixing the blood smears, the prepared slides were stored in special boxes containing cold accumulators and humidity controllers. Blood samples on slides, accompanied by the transmission sheets, were regularly collected in the field and transported to the parasitology laboratory of the “Institut National d’Hygiene (INH)” to be registered, checked, and analysed. After being stained with Giemsa, the slides were examined for the presence of the parasite. Each slide was analysed independently and blindly by two different biologist technicians. In case of discrepancies between the results of the two technicians, the slide was re-examined by a senior biologist technician [16].

### Study variables

The dependent variable in this study is the infection status of the child (positive/negative) on the microscopic examination of the malaria parasitaemia. The main independent variable was the region. Other independent variables included environment related factors such as altitude, household density, main water source, type of toilet facility, main material of floors, main material of walls and main material of roof. Human related factors included household wealth quintiles, age, gender, possession and use of a mosquito bed net, mother's education level, knowing mosquitoes as vectors of malaria, being exposed to malaria prevention messages, as well as ethnicity, religion and child had fever in last two weeks before the study. The household wealth quintile variable was constructed using principal component analysis on household’s assets and amenities [17]. A standardized composite measure combining household assets and possessions primarily based on selected assets, such as televisions and bicycles, materials used for housing construction, and types of water access and sanitation facilities was used [18].

### Data analysis

For each of the factors recorded, we assessed associations with malaria infection in univariate logistic regression using Generalized Linear Models (GLM), calculating odds ratios. This choice is explained by the fact that the GML is a very reasonable and general approach which consists in using variables (x's) to estimate the probability p that y = 1 [19]. Any association that was found to be statistically significant at a level of p ≤ 0.10 in univariate analysis was assessed as a potential confounder which could explain the observed heterogeneity in the multivariate analysis. In multivariable model, region of residence and all the potential confounders as well as any other factors that were significantly associated in univariate analysis (p ≤ 0.10) were included. Each of the secondary exposures were then removed one at a time, starting with the one with the highest p-value. If this resulted in a change of more than 10% in the odds ratio of region or if the likelihood ratio test comparing the complex model with the simple model was significant, the secondary exposure was retained. This process was continued until all remaining secondary exposures were either important as confounders or their removal would result in a significantly less precise model [20]. The Interactions test between our main variable of interest (region of residence) and each of the secondary exposures was checked. For this purpose, categorical variables with more than two levels were recoded to binary. Dose response curves were generated using the predictor effect plots [21] to show the trend between the malaria prevalence and certain associated risk factors. Data were analysed using the software R version 4.0.4.

## Results

### Characteristics of the study population

A total of 3,271 children aged between 0 and 59 months from 5,130 households were enrolled in the survey, of whom 2131 aged 6–59 months were tested for the presence of malaria parasites. There were almost equal numbers of female and male children, with a median age of 27 months (IQR 13–43). Most children (66.7%) were from rural areas, except for the Lomé Commune region, which is an urban agglomeration that includes the country's capital. The largest group of children in our sample (24.1%) were from the poorest quintile of households, while only 16.6% were from the richest quintile (Table 1). In addition, most of the children were living in houses where the main materials of the floor, wall and roof were not upgraded (Table 1).

**Table 1:** Characteristics of the study population

Factor	Number (%)
Health Region	
Lomé Commune	616 (20.7)
Maritime	531 (17.9)
Plateaux	764 (25.7)
Centrale	290 (9.8)
Kara	328 (11.0)
Savanes	443 (14.9)
Place of residence	
Rural	1983 (66.7)
Urban	989 (33.3)
Gender	
Male	1480 (49.8)
Female	1492 (50.2)
Age in months	
06–23	1022 (34.4)
24–35	616 (20.7)
36–59	1334 (44.9)
Had fever in last two weeks	
No	2243 (75.5)
Yes	729 (24.5)
Mother’s ethnicity	
Kabye_tem	895 (30.1)
Mina	997 (33.5)
Para-gourma	896 (30.1)
Stranger	89 (3.0)
Other	95 (3.2)
Mother’s religion	
Animist	836 (28.1)
Christian	1564 (52.6)
Muslim	567 (19.1)
Other	55 (0.2)
Mother’s educational level	
No education	1269 (42.7)
Primary	993 (33.4)
Secondary or higher	710 (23.9)
Mother's age in years	
15–24	734 (24.7)
25–34	1464 (49.3)
35–49	774 (26.0)
Exposed to malaria messages	
No	2293 (77.2)
Yes	679 (22.8)
Recognising mosquitoes as vectors of malaria	
No	575 (19.3)
Yes	2397 (80.7)
Household has mosquito bed net	
No	174 (5.9)
Yes	2798 (94.5)
Household wealth quintile	
Poorest	715 (24.1)
Poorer	645 (21.7)
Middle	581 (19.5)
Richer	538 (18.1)
Richest	493 (16.6)
Main floor material	
Improved	2465 (82.9)
Unimproved	507 (17.1)
Main wall material	
Improved	1758 (59.2)
Unimproved	1214 (40.8)
Main roof material	
Improved	2534 (85.3)
Unimproved	438 (14.7)
Main water source	
Improved	1827 (61.5)
Unimproved	1145 (38.5)
Type of toilets facility	
Improved	1166 (39.2)
Unimproved	1806 (60.8)
Density in the household	
1–2 persons	34 (1.2)
3–4 persons	658 (22.1)
5 + persons	2280 (76.7)
Altitude in metres	
0–300	2179 (73.3)
301–600	749 (25.2)
601 and more	44 (1.5)

**Fig. 3:**
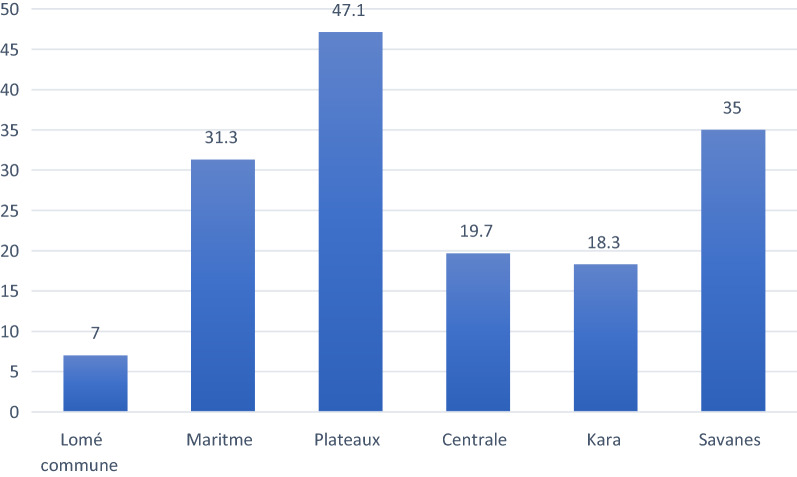
Malaria prevalence in children by region in Togo in 2017

Children living in the Plateaux region had the highest prevalence of malaria (47.1%) followed by children living in the Savanes (35.0%) and Maritime (31.3%) regions. Intermediate prevalence levels of malaria were recorded in Kara (18.3%) and Centrale (19.7%), and the lowest prevalence level (7.0%) in Lomé region (Fig. 3; Table 2).

**Table 2:** Crude and adjusted risk factors for malaria parasitaemia in Togo children

Factors	Positive malaria test* (n = 841)	Negative malaria test* (n = 2131)	OR (95% CI)	aOR (95% CI)
	N (%)	N (%)		
Health region				
Lomé Commune	43 (7.0)	573 (93.0)	Ref	Ref
Maritime	166 (31.3)	365 (68.7)	6.9 (4.5–11.1)	2.0 (1.1–3.6)
Plateaux	360 (47.1)	404 (52.9)	13.7 (8.9–21.8)	4.2 (2.4–7.6)
Centrale	57 (19.7)	233 (80.3)	3.7 (2.4–6.2)	1.9 (0.9–3.6)
Kara	60 (18.3)	268 (81.7)	3.1 (1.9–5.0)	1.1 (0.5–1.9)
Savanes	155 (35.0)	288 (65.0)	7.7 (5.0–12.2)	1.5 (0.8–2.8)
Place of residence				
Urban	738 (37.2)	1245 (63.8)	Ref	Ref
Rural	103 10.4)	886 (89.6)	5.19 (4.0–6.8)	2.02 (1.3–3.1)
Child's gender				
Male	428 (28.9)	1052 (71.1)	Ref	Ref
Female	413 (27.7)	1079 (72.3)	0.95 (0.8–1.1)	-
Age in months				
06–23	196 (19.2)	826 (80.8)	Ref	Ref
24–35	177 (28.7)	439 (71.3)	1.42 (1.02–1.99)	1.4 (1.1–1.8)
36–59	468 (35.1)	866 (64.9)	1.90 (1.37–2.66)	2.5 (2.0–3.1)
Had fever in last two weeks				
No	139 (6.2)	2104 (93.8)	Ref	Ref
Yes	702 (96.3)	27 (3.7)	1.48 (1.17–1.87)	1.1 (0.8–1.4)
Mother’s ethnicity				
Kabye_tem	221 (24.7)	674 (75.3)	Ref	Ref
Mina	278 (27.9)	719 (72.1)	1.13 (0.91–1.39)	0.9 (0.6–1.2)
Para-gourma	240 (26.8)	656 (73.2)	1.26 (1.01–1.57)	1.1 (0.8–1.3)
Stranger	20 (22.5)	69 (77.5)	0.75 (0.44–1.24)	0.8 (0.8–1.2)
Other	82 (86.3)	13 (13.7)	2.85 (2.03–4.01)	1.2 (0.9–2.4)
Mother’s religion				
Animist	359 (42.9)	477 (57.1)	Ref	Ref
Christian	349 (22.3)	1215 (77.7)	0.5 (0.4–0.6)	0.7 (0.5–1.0)
Muslim	130 (22.9)	437 (77.1)	0.4 (0.3–0.5)	0.8 (0.6–1.1)
Other	3 (60.0)	2 (40.0)	0.4 (0.2–2.67)	0.6 (0.4–0.9)
Mother’s educational level				
No education	488 (38.5)	781 (61.5)	Ref	Ref
Primary	261 (26.3)	732 (73.7)	0.62 (0.5–0.7)	0.8 (0.63–0.9)
Secondary or higher	92 (13.0)	618 (87.0)	0.27 (0.2–0.3)	0.6 (0.5–0.8)
Mother's age in years				
15–24	174 (23.7)	560 (76.3)	Ref	Ref
25–34	435 (29.7)	1029 (70.3)	1.08 (0.8–1.3)	1.2 (0.8–2.0)
35–49	232 (30.0)	542 (70.0)	1.33 (1.1–1.7)	1.4 (0.9–2.1)
Exposed to malaria messages				
No	689 (30.0)	1604 (70.0)	Ref	Ref
Yes	152 (22.4)	527 (77.6)	0.6 (0.4–0.7)	0.67 (0.5–0.8)
Recognising mosquitoes as vectors of malaria				
No	207 (36.0)	368 (64.0)	Ref	Ref
Yes	622 (25.9)	1775 (74.1)	0.55 (0.46–0.68)	0.9 (0.7–1.8)
Household has mosquito bed net				
No	25 (14.4)	149 (85.6)	Ref	Ref
Yes	816 (29.2)	1982 (70.8)	4.27 (2.49 7.98)	1.3 (0.9–2.1)
Household wealth quintile				
Poorest	359 (50.2)	356 (49.8)	Ref	Ref
Poorer	248 (38.4)	397 (61.6)	0.5 (0.4–0.7)	0.8 (0.7–1.1)
Middle	146 (25.1)	435 (74.9)	0.4 (0.4–0.6)	0.6 (0.5–0.8)
Richer	72 (13.4)	466 (86.6)	0.3 (0.2–0.4)	0.6 (0.4–1.0)
Richest	16 (3.2)	477 (96.8)	0.2 (0.1–0.2)	0.2 (0.1–0.4)
Type of toilet facility				
Improved	179 (15.4)	987 (84.6)	Ref	Ref
Unimproved	662 (36.7)	1144 (63.3)	2.56 (2.12–3.09)	2.1 (0.9–3.0)
Main roof material				
Improved	643 (25.4)	1891 (74.6)	Ref	Ref
Unimproved	198 (45.2)	240 (54.8)	5.80 (3.80–9.12)	1.4 (0.8–2.5)
Main wall material				
Improved	394 (22.4)	1364 (77.6)	Ref	Ref
Unimproved	447 (36.8)	767 (63.2)	1.74 (1.48–2.05)	1.2 (0.6–1.9)
Main floor material				
Improved	614 (24.9)	1851 (75.1)	Ref	Ref
Unimproved	227 (44.8)	280 (55.2)	2.16 (1.78–2.63)	1.1 (0.9–1.9)
Main water source				
Improved	429 (23.5)	1398 (76.5)	Ref	Ref
Unimproved	412 (36.0)	733 (64.0)	1.86 (1.58–2.19)	1.5 (0.7–2.1)
Density in the household				
1–2 persons	5 (14.7)	29 (85.3)	Ref	Ref
3–4 persons	158 (24.0)	500 (76.0)	2.34 (0.80–9.99)	1.9 (0.8–3.0)
5 + persons	678 (29.7)	1602 (70.3)	3.41 (1.18–14.41)	2.7 (0.9–5.8)
Altitude in metres				
0–300	718 (33.0)	1461 (67.0)	Ref	Ref
301–600	112 (15.0)	637 (85.0)	0.41 (0.3–0.5)	0.45 (0.3–0.6)
601 and more	11 (25.0)	33 (75.0)	0.37 (0.1–0.8)	0.23 (0.1–0.5)

*OR* Crude OR, *aOR* Adjusted odds ratio for all other factors considered in the multivariate analysis., *Ref* reference modality

*Results of the malaria test performed under the microscopy

### Regional heterogeneity of malaria in Togo

The region of residence was significantly associated with the child's malaria status (Table 2). Persistence of regional heterogeneity was explored following correction for the study variables described in the methods. Then, the Plateaux and Maritime regions remained at increased risk compared to Lomé, while the other regions no longer were at increased risk. The probability of a positive malaria test for children living in the Plateaux decreased from 13.7 times higher risk to 4.2 times higher risk compared to those living in the Lomé Commune region. (see Table 2). The same observation was made for the Maritime region, where the probability of a positive malaria test decreased from 6.9 times higher risk to 2 times higher risk compared to those living in the Lomé Commune region.

### Other significant risk factors related to increased malaria prevalence among children

**Fig. 4:**
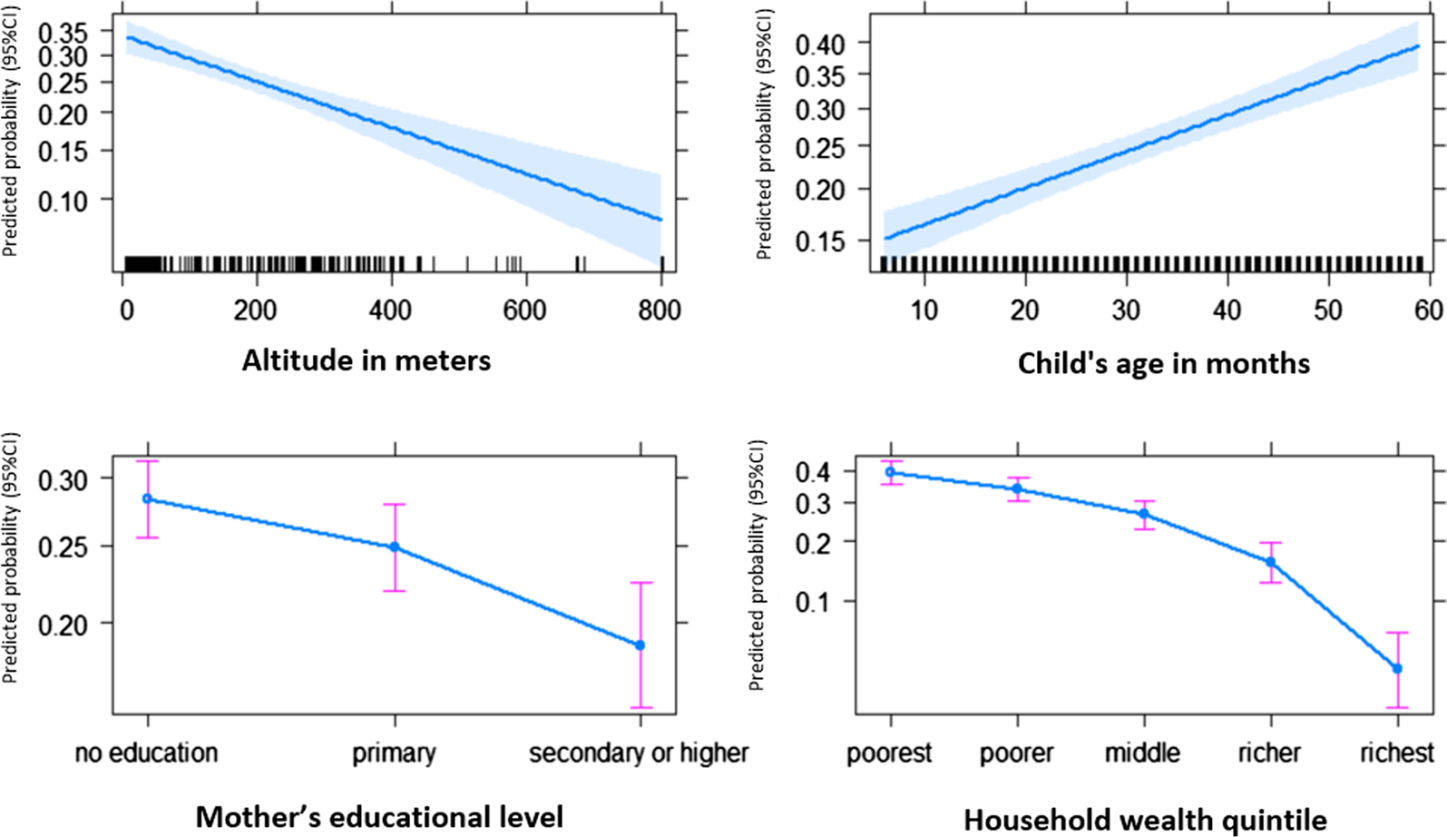
Predicted probabilities of malaria on altitude, child age, mother educational level and household wealth quintile

Table 2 summarized variables that had an independent statistically significant impact on the malaria prevalence. Children living in rural areas were at twice as high risk (adj. OR = 2.02, 95% CI [1.32–3.13]) to test positive for malaria compared to those living in urban areas. Malaria prevalence was highest among children whose mother had no education (38.5%), followed by those whose mother had primary education (26.3%), and finally those whose mother had secondary education and above (13.0%), showing a clear dose–response association (Fig. 4). Child age, household wealth quintile, altitude also showed an dose–response trend (Fig. 4). The probability of malaria in children decreased with increasing altitude, maternal education, and household wealth, while malaria prevalence increased with child’s age in months (Fig. 4). The interaction tests were not significant.

## Discussion

The prevalence of malaria in Togo in children under 5 years of age was high and varied by region, with the highest prevalence in the Plateaux region. After adjusting for several known risk factors, the effect of region of residence decreased but remained significant. This suggests that the repeatedly observed heterogeneity by region of residence reflects residual confounding by other variables not considered in the study. Such variables could include temperature, humidity, the presence of standing water in the household, the proximity of the household to a body of water, availability and quality of health care. The persistence of a higher prevalence in two of the six regions, Plateaux and Maritime, could potentially be explained by climatic conditions favourable for high mosquito densities in this region. The high rainfall provides an excellent breeding ground for the Anopheles mosquito [22]. Entomological studies in Nigeria [23] and Eritrea [24], also showed higher number of mosquitoes in the irrigated community compared to those in the non-irrigated community.

The observed increased risk of malaria for children living in rural areas may also be associated with more favourable conditions for mosquito development. Studies in Ghana [25] and Burkina Faso [26] found that proximity of dwellings to agricultural areas and rural water reservoirs increased the risk of malaria morbidity due to greater exposure to malaria vectors. The difference in risk between rural and urban children in our study was greater in the Lomé Commune region, the most urbanized region in Togo. Urbanization reduces the risk of malaria because it creates an environment which is suboptimal for mosquito persistence as it reduces the number of breeding sites [25, 26]. Urbanization also changes health practices, which can result in more effective prevention (use of insecticides, mosquito nets, preventive treatments), information dissemination (concentration of public and private media), and better access to health facilities [27, 28]. The difference in malaria risk by household wealth quintile suggests that malaria risk depends on wealth quintile [29, 30]. In contrast to our study, similar studies in Uganda [31] and Kenya [32], did not observe an association between household wealth index and malaria prevalence. This can be explained by the fact that these two studies were conducted in single rural communities, rather than nationwide including both urban and rural populations and a mix of different communities.

The higher malaria prevalence in older age groups observed in our study is consistent with observations in Kenya [33] and Tanzania [34]. An explanation could be that younger children have anti-malaria antibodies that have been transmitted to them by their mothers [34, 35]. Or that they spent less time outdoors in the evenings compared to older children, resulting in less exposure and are likely to be put under bed nets regularly [36]. Other explanations may include the fact that they have received Seasonal Malaria Chemo-prevention (SMC). This could guide considerations of more targeted malaria control strategies for older children, such as extending the age group covered by SMC.

Children living in areas with high malaria transmission intensity develop immunity with age because of continuous exposure to infected mosquito bites [37]. This immunity develops first against severe forms of malaria and then against non-severe malaria [35]. This could explain that older children are more likely to have malaria parasites without developing clinical disease, in contrast to younger children who still have less mature immunity and therefore still struggle more with malaria infections [35]. A mother's educational level showed a protective effect against malaria infection for her child. The same results were found using cross-sectional survey data from three African countries (Angola, Tanzania, Uganda) [38] and also in Democratic Republic of Congo [39]. An educated mother in general has a higher capacity for self-reliance and to break with certain traditions and beliefs that are not favourable to health. She is also more likely to be able to keep the household healthy and improve hygiene [40]. This positive effect of a mother's education on child survival is true even in poor households [41, 42]. Indeed, educated mothers are known to be more receptive to awareness raising campaigns to prevent children from getting sick, and are quicker to consult and use health services [42].

The dataset used for these analyses was derived from a cross-sectional survey and therefore has some limitations. For example, a causal relationship between the explanatory variables and the prevalence of malaria in children under five cannot be established. Also, data were collected on adherence to the first dose of seasonal malaria chemoprevention (SMC) drug in 2017. SMC was only available in three regions and 1061 children were exposed to the treatment as reported by caregivers with 99.9% adherence during the survey. Because of this low number of children exposed to SMC in the study sample, this were unable to control for this variable in the analyses. The large and nationally representative study population is an important strength of the study. Another strength of the study is that the malaria infection was confirmed by microscopy, which is the gold standard. In addition, the use of Generalized Linear Models allowed explaining part of the regional heterogeneity of malaria prevalence in under-five in Togo and the associated risk factors. This information can thus be used in the design of appropriate interventions.

## Conclusion

This study showed a strong regional heterogeneity of malaria prevalence in children under five years of age in Togo. Part of this heterogeneity could be explained by factors such as place of residence, child’s age, mother's education level, household wealth quintile, exposure to malaria prevention messages and altitude, but after adjusting for these, significant heterogeneity persisted. These findings support designing targeted interventions and strategies tailored to older children aged more than 24 months living in rural areas and from households with a poor wealth index. Particular emphasis could be placed on designing key malaria prevention messages for the high malaria prevalence regions. Prioritizing high prevalence regions for SMC and the combined use of preventive measures is recommended for better control of malaria in Togo.

## Data Availability

DHS datasets are publicly available on www.dhsprogram.org.

## Abbreviations

CHW: Community Health Workers
CBRS: “Comité Bioéthique de Recherche en Santé
DALY: Disability-Adjusted Life Years
EA: Enumeration Areas
GLM: Generalized Linear Models
INSEED: National Institute of Statistics
IRS: Indoor Residual Spraying
LLIN: Long-lasting Insecticidal Nets
PNLP: National Malaria Control Program
RDT: Rapid Diagnostic Test
SMC: Seasonal Malaria Chemo-prevention
TMIS: Togo Malaria Indicator Survey
WHO: World Health Organization
